# Study on the Impact of Cross-Reactive Antibodies Induced by SARS-COV-2 Infection on the False-Positive Rate of HIV Serological Testing

**DOI:** 10.1007/s12288-025-02081-0

**Published:** 2025-08-06

**Authors:** Dong Chen, Jiayi Zhang, Feng Zhang, Bi Lin, Jie Yang, Tong Chen, Ledan Chen, Chenwei Zhang, Fan Zhou

**Affiliations:** 1 Wenzhou Central Hospital, The Ding Li Clinical Institute of Wenzhou Medical University, Wenzhou, China; 2 School of Laboratory Medicine and Life Science, Wenzhou Medical University, Xueyuan West Road & 270#, Wenzhou, 325000 Zhejiang Province China; 3 Wenzhou Central Blood Station, Wenzhou, China; 4 The Second Affiliated Hospital of Shanghai University, Baili East Road &252#, Wenzhou, 325000 Zhejiang Province China; 5 Wenzhou Key Laboratory of Diagnosis and Treatment of Emerging and Recurrent Infectious Diseases, Wenzhou, China; 6 Wenzhou Key Laboratory of Blood Transfusion Medicine, Wenzhou, China

**Keywords:** SARS-COV2, COVID 19 pandemic, Non-remunerated blood donors, False reactivity, HIV serological detection

## Abstract

**Purpose:**

This study evaluates COVID-19 pandemic phase-specific variations in HIV seroprevalence among volunteer blood donors to elucidate determinants of assay discordance.

**Methods:**

This retrospective cohort study analyzed 681,232 volunteer blood donations (Wenzhou, 2017-2023) across four pandemic phases: pre-pandemic, containment, surge (Jan-Feb 2023), and stabilization. All specimens underwent dual HIV serological/nucleic acid screening, with reactive cases referred for CDC confirmation.

**Results:**

Analysis of 681,232 specimens revealed an overall HIV seroreactivity rate of 0.14%, escalating from 0.08% (pre-pandemic) to 0.91% during the Omicron surge phase (Jan-Feb 2023). Among 184 reactive cases in the surge period, 178 (96.7%) showed single-reagent reactivity with predominantly low S/CO ratios (94.9% <5.0), all Western blot (WB)-negative. Significant variance occurred across gender (P=0.043), age (P=0.007), and donation frequency (P=0.001). Indeterminate WB results (n=37) exhibited 5 immunoblot band patterns, with P24 predominance suggesting assay interference.

**Conclusion:**

SARS-CoV-2 infection induces HIV false-positive seroreactivity, necessitating algorithm optimization in clinical laboratories during pandemic waves to conserve blood resources.

## Introduction

Acquired immunodeficiency syndrome (AIDS)is a severe infectious disease transmitted primarily through sexual, vertical, and bloodborne routes [1]. The human immunodeficiency virus (HIV) specifically targets lymphocytes within the human immune system, particularly CD4+T cells, whose depletion constitutes a major threat to host immune competence [2]. Transfusion of HIV-contaminated blood products carries significant infection risks, making accurate detection of HIV antigens/antibodies in blood products a critical component of transfusion safety protocols [3]. However, inter-manufacturer variability in ELISA kit production processes, including coating materials and methodological approaches, may generate false-positive(FP) reactivity [3–5]. Such FP results lead to permanent deferral of eligible non-remunerated blood donors, resulting in both substantial blood resource wastage and unnecessary psychological distress for affected donors and their families. Minimization of FP reactivity remains a persistent operational challenge for blood collection services worldwide. The COVID-19 outbreak in late 2019 significantly impacted global public health security. Following dynamic adjustments to China's epidemic prevention policies, the nationwide lifting of COVID-19 control measures on December 7, 2022 led to widespread community transmission. Limited data exist regarding potential effects of SARS-CoV-2 infection recovery on HIV serological markers and nucleic acid test results among non-remunerated blood donors. This study systematically analyzed HIV serological test results from non-remunerated blood donors across distinct pandemic phases. Our objectives were to: (1) identify factors influencing post-COVID-19 HIV serological testing outcomes, and (2) establish evidence-based guidelines for healthcare institutions, blood collection services, and public health agencies. The findings aim to optimize testing protocols and resource allocation in post-pandemic healthcare settings.

## Materials and Methods

1. Experimental Subjects

A total of 681,232 non-remunerated blood donors in Wenzhou from January 2017 to November 2023 were included. They were categorized based on different periods of the COVID-19 pandemic: pre-pandemic period (2017–2019): 299,239 individuals; infection control period (2020–2022): 330,578 individuals; concentrated infection period (January–February 2023): 20,170 individuals; infection stable period (September–November 2023): 31,245.

2. Equipment and Reagents

Detection Equipment: TECAN Freedom EVO 150/8 Fully Automated Liquid Handling System (TECAN), Microlab FAME Fully Automated Enzyme Immunoassay Processing System (F.A.M.E.24/20, Hamilton Bonaduz AG), Sunrise Microplate Reader (TECAN), Cobas Nucleic Acid Testing System (Roche, USA).

Serological Detection Reagents (Enzyme-Linked Immunosorbent Assay, ELISA): Reagent 1: Human Immunodeficiency Virus Antibody Diagnostic Kit (Inno Diagnostics), Reagent 2: Human Immunodeficiency Virus Antigen–Antibody Diagnostic Kit (Bio-Rad, France).

Nucleic Acid Testing Reagents (PCR-Fluorescence Method): HBV-HCV-HIV (1 + 2 Types) Nucleic Acid Test Kit (Roche Diagnostics).

### Serological Detection

  Serum specimens from non-remunerated blood donors were screened using ELISA kits from two distinct manufacturers. The HIV serological status was determined according to the following algorithm: Negative result: Both kits demonstrated non-reactivity. Double-reactive result: Both kits showed reactivity or indeterminate reactivity. Confirmatory testing: When either kit produced reactive or indeterminate results, duplicate testing was performed using the reactive kit. Single-reactive result: Only one of the duplicate wells exhibited reactivity or indeterminate reactivity.

### Nucleic Acid Testing

Specimens from non-remunerated blood donors were tested for HIV-RNA using a pooled nucleic acid testing (NAT) approach with a pool size of six. The testing algorithm was implemented as follows: Initial pooled testing: Non-reactive pool result: All six specimens were classified as HIV-RNA negative. Reactive pool result: Individual NAT was performed on each constituent specimen. Individual testing: All non-reactive individual results: Final classification as HIV-RNA negative. Any reactive individual result: Final classification as HIV-RNA positive.

### Confirmatory Testing

For specimens that showed reactivity in serology and/or nucleic acid testing, they were sent to the Wenzhou CDC following biosafety packaging requirements for HIV WBconfirmatory testing. Our study employed WB as the gold standard confirmatory test for HIV diagnosis both before and during the COVID-19 pandemic. As detailed in Supplementary Table 1, WB confirmatory testing was routinely performed on all initially reactive specimens throughout the pre pandemic period (2017-2019), demonstrating consistent implementation of this confirmatory protocol.

### Statistical Analysis

Statistical processing involved the use of SPSS 20.0 statistical software for data analysis, and the comparison of reactive rates was conducted through the chi-square (*X*^*2*^) test. A significance level of *P* < 0.05 was considered to indicate statistically significant differences.

## Results

### Serological Detection Results of HIV in Non-remunerated Blood Donors During Different Phases of the COVID-19 Pandemic

During different phases of the COVID-19 pandemic, HIV serological detection results among non-remunerated blood donors were examined. From January 2017 to November 2023, a total of 681,232 specimens were detected, with 957 reactive specimens, yielding a reaction rate of 0.14%. In the pre-infection phase of the COVID-19 pandemic, 299,239 specimens were detected, resulting in 249 reactive specimens (0.08% reaction rate). The reaction rates for individual detects were 0.01% for reagent 1, 0.06% for reagent 2, and 0.01% for both reagents. The pre-infection phase demonstrated a confirmed positivity rate of 0.013% (39 WB-confirmed positives among 299,239 specimens).

During the infection control phase, 330,578 specimens were detected, leading to 435 reactive specimens (0.13% reaction rate). The individual detect reaction rates were 0.03% for reagent 1, 0.09% for reagent 2, and 0.01% for both reagents. The infection control phase exhibited a significantly lower WB confirmed HIV positivity rate (0.009%) compared to the pre-pandemic period (0.013%), with pronounced false reactivity in 2021 likely linked to pandemic-related immunological interference. In the concentrated infection phase, 20,170 specimens were detected, resulting in 184 reactive specimens (0.91% reaction rate). Reagent 2 exhibited a notably elevated reaction rate during this phase, being 9.8 times higher than the infection control period and 14.7 times higher than the pre-infection phase.Despite a high reactive specimen rate (0.91%), the WB confirmation rate was extremely low (0.005%). In the infection stabilization phase, 31,245 specimens were detected, leading to 89 reactive specimens (0.28% reaction rate).Similar to the concentrated infection phase, Reagent 2 dominated reactivity, but only 1 case was confirmed. For detailed HIV serological detection results during different phases, refer to Table 1.

**Table 1:** Serological detection results of HIV in non-remunerated blood donors during different phases of the COVID-19 pandemic

Different phases of the COVID-19 pandemic	Years	Total	Reactive specimens n(%)	Reaction rate for reagent 1 n(%)	Reaction rate for reagent 2 n(%)	Reaction rate for both reagents n(%)	WBconfirme d positives	Confirme d positivity rate(%)
Pre-infection phase	2017	95,072	64(0.07%)	9(0.01%)	44(0.05%)	11(0.01%)	11	0.0
	2018	97,693	85(0.09%)	17(0.02%)	53(0.05%)	15(0.02%)	15	0.0
	2019	106,474	100(0.09%)	15(0.01%)	72(0.07%)	13(0.01%)	13	0.0
Infection control phase	2020	105,357	106(0.10%)	19(0.02%)	74(0.07%)	14(0.01%)	14	0.0
	2021	109,786	150(0.14%)	55(0.05%)	89(0.08%)	6(0.01%)	7	0.0
	2022	115,435	179(0.16%)	26(0.02%)	143(0.12%)	10(0.01%)	10	0.0
Concentrated infection phase	January to February 2023	20,170	184(0.91%)	5(0.02%)	178(0.88%)	1(0.01%)	1	0.0
Infection stabilization phase	September to October 2023	21,111	75(0.36%)	1(0.01%)	74(0.35%)	0(0.00%)	1	0.0
	November 2023	10,134	14(0.14%)	2(0.02%)	11(0.11%)	1(0.01%)	0	0.0

### Single Reagent 2 Reactive Specimen WB Confirmatory Testing

In January and February 2023, a total of 20,170 specimens were detected for the reactivity of single reagent 2. Among the 178 reactive specimens, 169 had S/CO values in the range of 0.85–5.0, accounting for 94.9%. Nine specimens had S/CO values between 5.0 and 10.0, constituting 5.1%. Of the 178 specimens, 141 detected negative in the WB Confirmatory testing conducted by the Wen Zhou CDC, representing 79.2%. There were no reactive results, and 37 specimens yielded uncertain results, making up 20.8%. Refer to Table 2 for detailed information on WB confirmatory testing results of reactive specimens from single reagent 2.

**Table 2:** WB confirmatory testing results of reactive specimens from single reagent 2

Years	Total (n = 20,170)	Number of specimens submitted for testing (n = 178)	Reactivity of single reagent 2		NAT reactivity count	HIV WB confirmatory testing		
			S/CO value 0.85–5.0 (n = 169)	S/CO value 5.0–10.0 (n = 9)		Negative (n = 141)	Reactive	Uncertain (n = 37)
January 2023(n, %)	7516(37.3%)	57(32%)	56(33.1%)	1(11.1%)	0	42(29.8%)	0	12(32.4%)
February 2023(n, %)	12,654(62.7%)	121(68%)	113(66.9%)	8(88.9%)	0	99(70.2%)	0	22(67.6%)

### Comparison of General Information of Single Reagent 2 Reactive Blood Donors

In January and February 2023, a total of 20,170 specimens were detected for the reactivity of the single reagent 2. Out of the 178 reactive specimens, 169 exhibited S/CO values within the range of 0.85–5.0, constituting 94.9%. Nine specimens had S/CO values between 5.0 and 10.0, accounting for 5.1%. Among these 178 specimens, 141 detected were negative in the WB confirmatory testing conducted by the CDC, representing 79.2%. There were no reactive results, and 37 specimens yielded uncertain results, making up 20.8%. Detailed information on the WB confirmatory testing results of reactive specimens from single reagent 2 is available in Table 3.

**Table 3:** Detailed information on the WB confirmatory testing results of reactive specimens from single reagent 2

Groups	Total	Non-reactive	Reactive	Reactivity (%)	χ^2^ value	*P* value
*Sex*						
Male	12,690	12,591	99	0.78	4.099	< 0.05
Female	7480	7401	79	1.06		
*Blood groups*						
A	5975	5930	45	0.75	4.691	0.196
B	5027	4989	38	0.76		
O	7652	7574	78	1.02		
AB	1516	1499	17	1.12		
*Age (years)*						
18–30	7744	7661	83	1.07	12.103	< 0.05
31–40	6740	6675	65	0.96		
41–50	4478	4453	25	0.56		
51–60	1208	1203	5	0.41		
*Donations frequency*						
One	8080	7984	96	1.19	14.698	< 0.05
2–3 times	5628	5587	41	0.73		
3 or more times	6462	6421	41	0.63		

### The WB Confirmatory Testing Results Show an Indeterminate Band Distribution and Reagent 2 S/CO Value Situation

The study conducted in January and February 2023 involved analyzing 20,170 specimens for single reagent 2 reactivity. Of the 178 reactive specimens, 94.9% had S/CO values between 0.85 and 5.0, while 5.1% ranged from 5.0 to 10.0. In WB confirmatory testing by the CDC, 79.2% detected negative, and 20.8% yielded uncertain results. Analysis of 178 reactive blood donors revealed a significant difference in reaction rates between genders (χ^2^ = 4.099, *P* = 0.043), no significant difference in blood types (χ^2^ = 4.691, *P* = 0.196), significant differences in age groups (χ^2^ = 12.103, *P* = 0.007), and donation frequency groups (χ^2^ = 14.698, *P* = 0.001). The WB confirmation of uncertain results included diverse band patterns and S/CO values, detailed in Table 4. General information of reactive blood donors from single Test 2.

**Table 4:** General information of reactive blood donors from single Test 2

		Single reagent 2 reactivity	
Band distribution	Total (n = 39)	S/CO value 0.85–5.0	S/CO value 5.0–10.0
P17(n, %)	10(27.0%)	7	3
P24(n, %)	7(18.9%)	6	1
P17 and P24(n, %)	3(8.1%)	3	0
P66(n, %)	5(13.5%)	4	1
P66 and P24(n, %)	4(10.8%)	4	0
P66 and P51(n, %)	3(8.1%)	3	0
Gp160(n, %)	4(10.8%)	4	0
Gp160 and P17(n, %)	1(2.7%)	1	0

## Discussion

Clinical blood transfusion therapy has emerged as a critical intervention for managing acute and critically ill patients, with its widespread clinical application in recent years accompanied by heightened scrutiny of blood product safety and transfusion practices [6]. Maintaining stringent quality control of blood products and ensuring transfusion safety constitute fundamental responsibilities of blood collection facilities. As mandated by the Technical Operating Procedures for Blood Stations (2019 Edition), comprehensive HIV screening must include at least one nucleic acid test (NAT) and one serological assay for infection markers [7]. However, operational challenges persist, with false-reactive results arising from multiple sources: Donor-related factors: including hematologic malignancies, autoimmune disorders, post-kidney transplantation status, and chronic renal failure, technical variables: encompassing specimen hemolysis, fibrinogen interference, and suboptimal incubation durations [8–12]; reagent limitations: particularly batch-to-batch variability and the inherent challenge of optimizing both sensitivity and specificity [13]. These complexities underscore the necessity of implementing robust, multi-stage quality assurance protocols throughout the blood screening process. In this study, HIV serological screening of non-remunerated blood donors was performed using two ELISA kits from different manufacturers. A gray zone threshold was set at 85% of the cut-off value, with any reactive result from either kit classified as an unacceptable serological outcome, leading to permanent deferral of the donor. The HIV seropositivity rates exhibited significant variation across different pandemic phases: 0.08% in the pre-pandemic period, 0.13% during the infection control period, and a marked increase to 0.91% in the concentrated infection period. Notably, the elevated seropositivity rate was primarily driven by an abnormal increase in reactivity with Kit 2. To rule out potential confounding factors related to specimens, personnel, or laboratory equipment, the laboratory team conducted a thorough internal audit. This included analysis of critical quality control parameters before, during, and after testing—covering personnel competency, instrument performance, reagent quality, methodological consistency, environmental conditions, and procedural integrity—none of which revealed any abnormalities. Additionally, all experimental procedures strictly adhered to the manufacturers' protocols, with optimized parameters for both ELISA kits. Despite these measures, Kit 2 continued to produce false-reactive results, suggesting an inherent limitation of the assay rather than an operational issue. The timeline of COVID-19 prevention and control in China can be categorized into distinct phases: the pre-pandemic period (2017-2019), the pandemic control phase (2020-2022), and the post-vaccination era beginning in early 2022. Following a major policy adjustment in December 2022 that led to the comprehensive lifting of restrictions, a surge of COVID-19 pneumonia cases emerged in the general population, temporarily disrupting non-remunerated blood donation activities. As recovered COVID-19 patients gradually resumed blood donation, many carried high-titer SARS-CoV-2 antibodies or non-specific substances that persisted for extended periods. The dramatic increase in reactive rates observed with Kit 2 likely resulted from cross-reactivity between these post-infection antibodies/non-specific substances and antigenic components in the test kit, leading to elevated false-positive results in HIV serological testing. This hypothesis was supported by the subsequent normalization of reactive rates when Kit 2 was temporarily replaced with Kit 3 in March 2023. Further evidence emerged when Kit 2 was reintroduced in September 2023, by which time antibody levels in the population had declined during the stable post-infection phase. The HIV reactive rate with Kit 2 progressively decreased from 0.88% during the concentrated infection period to 0.35% (September-October 2023) and further to 0.11% by November 2023, confirming that widespread SARS-CoV-2 infection following policy relaxation was the primary driver of Kit 2's abnormal performance. Demographic analysis of 178 reactive donors revealed higher false-positive rates among females, younger individuals, and first-time donors, potentially associated with differential antibody responses post-infection. WB confirmation testing showed limitations, with one-third of indeterminate results displaying the P24 band - a pattern suggesting possible false reactivity. These findings underscore the importance of continued monitoring and CDC follow-up for indeterminate cases, particularly those exhibiting the P24 band pattern. Following the complete lifting of social restrictions, a substantial increase in COVID-19 cases was observed, coinciding with elevated false-reactive rates in HIV serological testing and consequent wastage of valuable blood resources. This phenomenon has created not only psychological and lifestyle burdens for non-remunerated blood donors but has also emerged as a critical factor in the declining participation of blood donation initiatives. Our findings demonstrate that timely adjustment of laboratory testing protocols - through careful consideration of WB band patterns, ELISA reagent S/CO values, and nucleic acid test results during specific epidemiological periods - can effectively mitigate the psychological impact on affected donors while helping maintain stability in blood donor recruitment. Notably, such false-reactive phenomena have not been documented in international reports, despite the global use of similar testing reagents (Reagent 2). This discrepancy raises important questions regarding potential ethnic variations in post-infection nonspecific antibody production and the nature of COVID-19-related interference with HIV diagnostic assays. The absence of comparable reports internationally suggests either population-specific immunological responses or differences in testing algorithm implementation that warrant further investigation to clarify these unresolved scientific questions. This study identifies a critical public health challenge wherein SARS-CoV-2 infection/vaccination induces cross-reactive antibodies causing HIV seroassay interference, evidenced by a 14.7-fold increase in false-positive rates (0.91% reactivity; n=20,170) during China's 2023 Omicron surge using Kit 2. Current ethical deficiencies in donor management were exposed, including inadequate risk disclosure and inequitable permanent deferrals disproportionately affecting first-time (1.19%), young (1.07%), and female (1.06%) donors. Mitigation strategies - reagent substitution (Kit 2→3), tiered deferrals for low S/CO cases (<5.0; 94.9% of FPs), and rapid NAT integration - restored baseline reactivity (0.11% by Nov 2023). The absence of international reports despite global Kit 2 usage suggests potential ethnic immunological variations or algorithmic discrepancies, necessitating multinational cohorts to optimize assay validation. These findings advocate for pandemic-responsive diagnostics balancing accuracy with ethical donor management through transparent communication and adaptive protocols. The diagnostic landscape of HIV testing faces persistent challenges due to serological mimicry, where antibodies targeting unrelated infections cross-react with HIV antigens, generating false-positive results. Our research into SARS-CoV-2 interference revealed this phenomenon extends beyond coronaviruses, with implications for global testing protocols. During the COVID-19 pandemic, we observed how SARS-CoV-2 infection or vaccination could induce antibodies that bound weakly but detectably to HIV assay components, particularly in kits using whole viral lysates rather than recombinant antigens. This cross-reactivity, while typically producing lower signal-to-cutoff ratios than true HIV infection, nevertheless triggered unnecessary confirmatory testing and donor deferrals. The problem of serological mimicry is not new to HIV diagnostics. Historical data identify multiple pathogens capable of generating HIV cross-reactive antibodies, including malaria parasites in endemic regions, where Plasmodium falciparum infection has been shown to produce antibodies recognizing HIV-1 gp120. Similarly, chronic herpesvirus infections like EBV and CMV can stimulate polyclonal B-cell activation [14, 15], generating heterophilic antibodies that interfere with both screening and confirmatory assays. Autoimmune disorders (e.g., lupus-associated antinuclear antibodies) and whole-virus vaccines demonstrate cross-reactivity with HIV antigens, generating diagnostic interference. Our COVID-19-era analysis of >20,000 specimens revealed 0.91% false-positive (FP) rates, incurring substantial costs (WB confirmation: 5× screening expense) and donor attrition, particularly among first-time (FP rate 1.19%) and young donors (1.07%). Mitigation requires: 1) Algorithmic NAT prioritization for low-S/CO specimens; 2) Manufacturer-driven epitope optimization (e.g., removing conserved α-helix domains); 3) Tiered deferrals permitting retesting post-antibody decay. The pandemic underscores the necessity for adaptive diagnostic ecosystems integrating real-time epidemiological data with assay redesign, ensuring equity amid expanding immunogenic challenges. This study has key limitations: 1) Pandemic phase stratification may oversimplify SARS-CoV-2 immunological impacts on HIV cross-reactivity, compounded by lacking longitudinal donor data; 2) Cost-driven exclusion of ID-NAT prevents distinguishing viral humoral responses from assay interference; 3) Single-center design introduces geographical bias given regional variant predominance (BA.5/BF.7) and heterogeneous vaccination (inactivated/mRNA); 4) Absence of SARS-CoV-2 neutralizing antibodies/epitope data precludes mechanistic insights into structural protein involvement (S1-RBD vs nucleocapsid); 5) Indeterminate WB patterns (37/184) in low-avidity cases challenge conventional HIV-1/2

## Data Availability

All data generated or analyzed during this study are included in this article and its supplementary material files. Further enquiries can be directed to the corresponding author.

## References

[1] Okano JT, Sharp K, Valdano E, Palk L, Blower S (2020) HIV transmission and source-sink dynamics in sub-Saharan Africa. Lancet HIV 7:e209–e214 32066532 10.1016/S2352-3018(19)30407-2 PMC7259817

[2] Hendricks CL, Mellet J, Durandt C, Brittain D, Pepper MS (2023) Haematopoietic stem-cell transplantation in an HIV endemic area: time to consider donors exposed to or living with HIV. Lancet HIV 10:e742–e749 37837978 10.1016/S2352-3018(23)00198-4

[3] Hong Y, Huang X, Ling H, Liao H (2012) Prevalence and trend of HIV infection among voluntary blood donors in China since implementation of the Blood Donation Law: a systematic review and meta-analysis. Trop Med Int Health 17:978–988 22686403 10.1111/j.1365-3156.2012.03019.x

[4] Gostin L, Curran WJ (1987) AIDS screening, confidentiality, and the duty to warn. Am J Public Health 77:361–365 3544882 10.2105/ajph.77.3.361 PMC1646908

[5] Zaongo SD, Sun F, Chen Y (2021) Are HIV-1-specific antibody levels potentially useful laboratory markers to estimate HIV reservoir size? A review. Front Immunol 12:786341 34858439 10.3389/fimmu.2021.786341 PMC8632222

[6] Mueller MM, Van Remoortel H, Meybohm P, Aranko K, Aubron C, Burger R et al (2019) Patient blood management: recommendations from the 2018 Frankfurt Consensus Conference. JAMA 321:983–997 30860564 10.1001/jama.2019.0554

[7] Lynch RM, Bar KJ (2023) Development of screening assays for use of broadly neutralizing antibodies in people with HIV. Curr Opin HIV AIDS 18:171–177 37265260 10.1097/COH.0000000000000798 PMC11938708

[8] Ravindranath MH, Ravindranath RM, Morton DL, Graves MC (1994) Factors affecting the fine specificity and sensitivity of serum antiganglioside antibodies in ELISA. J Immunol Methods 169:257–272 7510761 10.1016/0022-1759(94)90270-4

[9] Bratcher PE, Gaggar A (2014) Factors influencing the measurement of plasma/serum surfactant protein D levels by ELISA. PLoS ONE 9:e111466 25365324 10.1371/journal.pone.0111466 PMC4218753

[10] Graham KM, Mylniczenko ND, Burns CM, Bettinger TL, Wheaton CJ (2016) Examining factors that may influence accurate measurement of testosterone in sea turtles. J Vet Diagn Invest 28:12–19 26699527 10.1177/1040638715618989

[11] Shang C, Wang Z, Liu H (2017) A terminal antibody method based on multiple factors that influence ELISA results for measurement of antibody affinity in clinical specimens. J Virol Methods 240:42–48 27889565 10.1016/j.jviromet.2016.11.009

[12] Revendova KZ, Zeman D, Bunganic R, Karasova K, Volny O, Bar M et al (2022) Serum neurofilament levels in patients with multiple sclerosis: a comparison of SIMOA and high sensitivity ELISA assays and contributing factors to ELISA levels. Mult Scler Relat Disord 67:104177 36130459 10.1016/j.msard.2022.104177

[13] Murphy G, Parry JV (2008) Assays for the detection of recent infections with human immunodeficiency virus type 1. Euro Surveill 13 18775293

[14] Tariq R, Pardi DS, Bartlett MG, Khanna S (2018) Low cure rates in controlled trials of fecal microbiota transplantation for recurrent clostridium difficile infection: a systematic review and meta-analysis. Clin Infect Dis 68:1351–1358. 10.1093/cid/ciy721 10.1093/cid/ciy721 30957161

[15] Shanks L, Klarkowski D, O'Brien DP (2013) False positive HIV diagnoses in resource limited settings: operational lessons learned for HIV programmes. PLoS One 8:e59906. 10.1371/journal.pone.0059906 23527284 10.1371/journal.pone.0059906 PMC3603939

